# Yoga and Emotion Regulation in High School Students: A Randomized Controlled Trial

**DOI:** 10.1155/2015/794928

**Published:** 2015-08-19

**Authors:** Leslie A. Daly, Sara C. Haden, Marshall Hagins, Nicholas Papouchis, Paul Michael Ramirez

**Affiliations:** ^1^Department of Psychology, Long Island University, Brooklyn Campus, 1 University Plaza, Brooklyn, NY 11201, USA; ^2^Department of Physical Therapy, Long Island University, Brooklyn Campus, 1 University Plaza, Brooklyn, NY 11201, USA

## Abstract

Middle adolescents (15–17 years old) are prone to increased risk taking and emotional instability. Emotion dysregulation contributes to a variety of psychosocial difficulties in this population. A discipline such as yoga offered during school may increase emotion regulation, but research in this area is lacking. This study was designed to evaluate the impact of a yoga intervention on the emotion regulation of high school students as compared to physical education (PE). In addition, the potential mediating effects of mindful attention, self-compassion, and body awareness on the relationship between yoga and emotion regulation were examined. High school students were randomized to participate in a 16-week yoga intervention (*n* = 19) or regular PE (*n* = 18). Pre-post data analyses revealed that emotion regulation increased significantly in the yoga group as compared to the PE group (*F* (1,32) = 7.50, *p* = .01, and eta^2^ = .19). No significant relationship was discovered between the changes in emotion regulation and the proposed mediating variables. Preliminary results suggest that yoga increases emotion regulation capacities of middle adolescents and provides benefits beyond that of PE alone.

## 1. Introduction

Emotion regulation is a growing area of interest for theorists and researchers alike, with adolescent studies steadily increasing over the past decade [1]. Thompson [2] defined emotion regulation as the “extrinsic and intrinsic processes responsible for monitoring, evaluating, and modifying emotional reactions, especially their intensive and temporal features, to accomplish one's goals.” Emotion regulation capacities are thought to be influenced by a range of systems in the body, including neurophysiological, physical, cognitive, behavioral, and social systems, and are particularly vulnerable during adolescence [3]. Developments in the physiological and neurological realms are often times at odds during adolescence, perhaps contributing to difficulties with emotion regulation, but the brain circuitry is also primed for specialization and novel skills [3–5]. Emotion dysregulation leads to a variety of negative outcomes and has been related to depression [6], self-injury [7], disordered eating, and drug and alcohol use in adolescents [8].

Yoga is a popular and comparatively inexpensive intervention that many schools are integrating into their curriculums to address an increasing need for self-regulatory skills such as emotion regulation. Significant results have been reported regarding improved anger management and impulse control, as well as a decrease in negative emotion for students in a yoga intervention [9–15], implying that perhaps some regulatory processes are indeed being impacted by yoga. However, only two studies have looked directly at emotion regulation as a construct [16, 17]. Using the Emotion Regulation Checklist (ERC) [18], Kokinakis [16] did not show any significant changes in emotion regulation for either a yoga or a control group. The yoga intervention in the study was spread out over time, which may have diluted its effects. Noggle et al. [17] found significant changes in subscales of the Difficulties in Emotion Regulation Scale (DERS) [19] which measured difficulties engaging in goal directed behavior and a lack of emotional awareness and an improvement, although not a significant change, in the overall score. While preliminary results suggest some shift in emotion regulation skills, the studies also highlight the importance of consistent dosing and using measures that capture the skills gained specifically through yoga (e.g., emotional awareness and decreased reactivity, in addition to emotion control and reframing). Research is still very much needed in this area and with this population, including urban, minority adolescents, a group contending with chronic stressors and the propensity to develop less secure attachments, and thus fewer opportunities to develop effective emotion regulation skills [20, 21].

In addition, research in this area is almost exclusively focused on efficacy with no efforts to examine mechanism [12, 23, 24]. However, more research is needed on the underlying mechanisms supporting potential benefits of yoga. Yoga is explicitly aimed at increasing mindful awareness, self-compassion, and body awareness and it is possible that these may serve as mediators for emotional well-being [22, 25, 26]. With a fuller understanding of underlying mechanisms and more clarity regarding which aspects of yoga influence what outcomes, yoga interventions may be more specifically designed to address the developmental needs of adolescents [16]. The purpose of the current study was to examine the effects of yoga on a group of middle adolescents' (15–17 years old [3, 4]) emotion regulation and to determine if mindful awareness, self-compassion, and body awareness contributed to those changes.

## 2. Materials and Methods

### 2.1. Participants

Prior to recruitment, Institutional Review Board (IRB) approval was obtained from Long Island University and the New York City Department of Education for this study. The study was conducted in a New York City public high school. A single school was selected due to an academic affiliation with the University, which provided increased logistical ease and access to the study participants. During the research design phase of the study, the school estimated that 75 students would be eligible for the study. With all of these participants, analyses running at 80% power would have been able to detect an effect size of at least 0.6 [31]. While it appeared promising that the study recruitment would yield a sample size close to the number of eligible participants, it was understood a priori that 100% recruitment was unlikely. Ultimately, only 62 students were eligible for the study. Given the lack of current evidence in this growing area of inquiry, we decided to move forward implementing the study with the understanding that there was a possibility of a limitation in power and thus potential for a type I error, which would be acknowledged and addressed as needed.

Inclusion criteria included being in good general health, as evidenced by permission to attend physical education (PE) class, and the ability to understand and answer questionnaires written in English. Participants were 38 students whose parents returned signed consent forms.

### 2.2. Yoga Intervention

Bent on Learning (BOL), a not-for-profit organization providing free yoga instruction in NYC classrooms, designed and implemented the 16-week yoga intervention [32]. Classes met on average three times per week, for 40 minutes, for 16 weeks. Changes in the school schedule (i.e., exam days and field trips) and holidays led to the cancellation of 6 classes. There were 42 sessions in all. Two teachers were present at every class. BOL's curriculum was created in accordance with the New York State and National Physical Education Standards. BOL teachers are required to have a 200-hour yoga certification and two years of teaching experience. Each class included postures, breathing, relaxation, and guided meditation techniques in a supportive and noncompetitive environment. The session included components of the following outline: a structured routine that transitioned the students into yoga, a rigorous 15–30-minute sequence of postures, relaxation, and a closing ritual that emphasized carrying principles of the yoga practice into the rest of the day [32]. The yoga class was observed three times to ensure consistency.

### 2.3. PE Control

The PE classes conformed to the New York State Department of Education Regulations. The learning standards for health and physical education focus on the knowledge and skills to establish and maintain physical fitness, participate in physical activity, and maintain personal health [33]. PE classes involved common games such as football and baseball as well as walking and running, relays, and other socially focused activities. PE was observed twice to ensure consistency.

### 2.4. Outcome Measure

Emotion regulation was assessed with the Emotion Regulation Index for Children and Adolescents (ERICA) [27]. The ERICA is a 16-item self-report measure that was completed by the participant. Rated on a five-point scale, the ERICA was found to be valid and reliable with a population of students in Australia [27]. Emotion regulation was further assessed by the Emotion Regulation Checklist (ERC) [18], a 24-item other-report measure designed to be completed by an adult that interacts with the particular student. The student's parent and teacher independently completed the measure. The measure is rated on a four-point scale and yields two subscales. The ERC was found to be valid and reliable with a sample of 513 parents of maltreated children and has been widely used [18]. A composite score, combining the students' total scores on the ERICA and total scores from the teachers and parents on the ERC, was intended to be used in the analyses.

### 2.5. Proposed Mediating Variables Measures

Mindful awareness was measured with the Mindful Attention Awareness Scale in Adolescents (MAASA) [28], a 14-item measure completed by the student. Rated on a 6-point scale, it measures a single factor and a core characteristic of mindfulness, the receptive state of mind, in which attention is focused on the events of the present moment (e.g., “I find it difficult to stay focused on what's happening in the present”). The measure is based on the adult version (MAAS) and was shown to be valid and reliable [28].

Self-compassion was assessed with the Self-Compassion Scale (SCS) [29], a 26-item self-report measure completed by the student. It is rated on a five-point scale and yields an overall self-compassion score in addition to six subscales. The overall score was used to represent the construct. The scale was validated on middle class, mostly Caucasian adolescents in Texas, and has excellent reliability [29].

The Multidimensional Assessment of Interoceptive Awareness (MAIA) [30] is a 32-item measure rated on a six-point scale and is completed by the student to assess body awareness. The measure has eight subscales. A total score was used to represent body awareness. In addition to discerning between an “anxiety-driven hypervigilance and a present-moment, mindful and accepting attention style to the same body sensations,” the scale is one of the first to represent the construct of body awareness on a multidimensional scale [30]. Validated on an adult population of mostly Caucasian subjects with some experience in mind-body modalities (i.e., yoga, Alexander technique, and Tai Chi), the measure was shown to be valid and reliable. Since the measure is new and had not been used with an adolescent population before, it was piloted with a group of students at the high school not involved in the study. Thirty-one high school juniors filled out the measure. The measure was reliable with this sample of the population and resulted in a Cronbach alpha of .88.

### 2.6. Procedure

Prior to asking for volunteers for the study, a research team member spoke with the students about stress and exercise, as well as yoga and research. The study was described to the students and they were invited to participate. Letters explaining the study, as well as consent for the child's participation, were handed out during parent-teacher conferences and given to students to bring home for the parents to fill out. An assent form for the child to fill out was also included. In addition, the parent was asked to complete a demographic form and the ERC. The parent or the child was asked to return the forms to the school. After this initial attempt to obtain parental consent, two more packets were sent home with the students over a two-month period. In addition, phone calls were made to remind the parents about the study and a research team member attended a parent meeting and presented the research project again to the parents in attendance.

Before the beginning of the 16-week intervention, at time 1, the participants were asked to fill out a packet of measures that included ERICA, MAASA, SCS, and MAIA. Physiological and attention measures were also taken at this time; however, the results from these measures are not included in this study. Teachers were asked to fill out the ERC. After this initial data collection session, participants were randomized using a computer program into either the yoga intervention or the PE class. Time 2 measures were administered at eight weeks. At this time, students, teachers, and parents were asked to fill out the same surveys as they did before the intervention began. Participants were assessed again within two weeks after the end of the intervention. The time 3, POST intervention assessment included the same measures as time 1 and time 2, as well as the physiological and attention measures. Due to a low return rate of the ERC from the teachers at time 1 and from the parents at all three time points, neither ERC nor time 2 was used in the analyses. Therefore, only the total score on the ERICA was used to represent the students' changes in emotion regulation rather than the composite score of the ERICA and the ERC from parents and teachers. Furthermore, this change left just two time points and a pre-post design (hereafter referred to as PRE and POST intervention).

## 3. Results

### 3.1. Demographics

**Figure 1:**
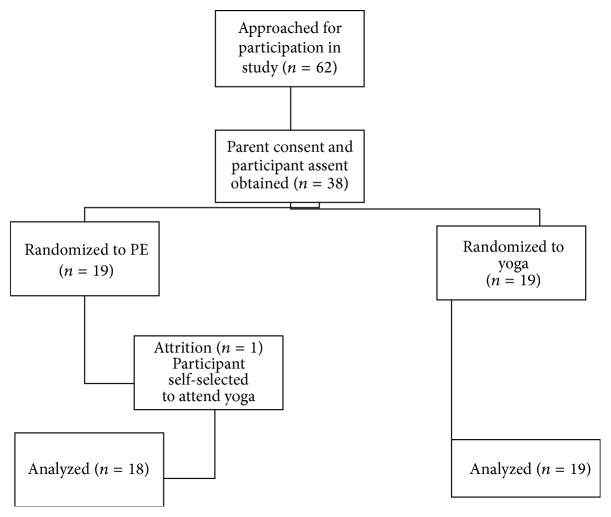
Participant flow.

Sixty-two students were eligible for the study. Thirty-eight (61%) signed parental consent forms were received. Assent was acquired by all 38 of these students. A computer program was used to randomly assign the students to either yoga or PE, with 19 participants in each group. One student self-selected yoga; however, and despite the research team's efforts to keep the student in PE, he attended yoga throughout the semester. This participant was removed from the analyses. Therefore, the groups were divided as follows: yoga (*n* = 19) and PE (*n* = 18) (Figure 1). No significant demographic or attendance differences were found using chi-square and *t*-test analyses.  Table 1 reports the demographic results.

### 3.2. Preliminary Analyses

All questionnaire and scale data were analyzed using SPSS Statistics, Version 20. All participants were included in the analyses, following intention-to-treat principles. Data were reviewed for return rates, missing data, and reliability issues (e.g., answering all items the same). Means and standard deviations were calculated for all measures and for change scores on the ERICA, MAASA, SCS, and MAIA. Scales were reviewed for normality, skewness, and kurtosis. All scales were normally distributed and skewness and kurtosis were less than 2.0 (Table 2). All scales were reliable at PRE and POST intervention, with Cronbach's alpha ranging from *α* = .70 to .90, with the exception of SCS at PRE with a Cronbach's alpha of *α* = .42.

### 3.3. Outcome Analysis

**Figure 2:**
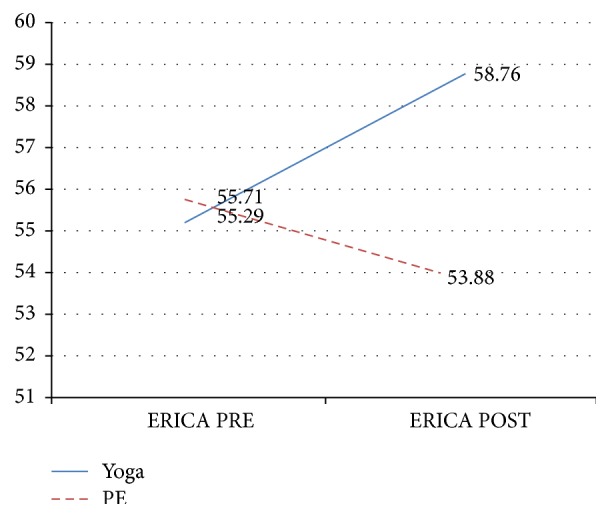
Emotion regulation (ERICA total mean scores) by group, PRE-POST intervention.* Note.* Yoga, *n* = 17, PE, *n* = 17; ERICA = Emotion Regulation Index for Children and Adolescents [22].

An ANOVA with the group assignment (PE or yoga group) as the between-subject variable was used to test the hypothesis that emotion regulation skills would increase in the yoga group. The analysis showed a significant interaction between time and group on emotion regulation, (*F*(1,32) = 7.50, *p* = .01, and eta^2^ = .19). Simple effects tests and univariate analyses of variance with emotion regulation (ERICA) at PRE and POST intervention as the dependent variable were conducted, but there was no main effect for group on emotion regulation PRE intervention (*F*(1,35) = .07, *p* = .79, and eta^2^ = .002) or POST intervention (*F*(1,32) = 3.21, *p* = .08, and eta^2^ = .09). However, inspection of the means revealed a crossover effect such that emotion regulation increased for the yoga condition and decreased for the PE condition. Specifically, emotion regulation at PRE intervention was greater for the PE condition (*M* = 55.71, SD = 5.95) than the yoga condition (*M* = 55.29, SD = 7.53) and at the POST intervention was greater for the yoga condition (*M* = 58.76, SD = 7.50) than the PE condition (*M* = 53.88, SD =8.36) (Figure 2).

### 3.4. Mediating Variable Analysis

Change scores, from PRE to POST intervention, were calculated for each of the potential mediating variable scales, MAASA (*n* = 34, *M* = .01, and SD = .86), SCS (*n* = 34, *M* = 1.12, and SD = 9.58), and MAIA (*n* = 32, *M* = .19, and SD = .64). Correlation analyses were run with change scores for ERICA (*n* = 34, *M* = .82, and SD = 6.17). Mindful attention and self-compassion were not significantly correlated with increases in emotion regulation. Body awareness (MAIA) change scores were significantly, positively correlated with emotion regulation (ERICA) change scores (*r* = .57, *p* < .01). Due to the nonsignificant findings between mindful awareness and self-compassion with emotion regulation, mediation analyses were not conducted.

## 4. Discussion 

Building upon previous studies [16, 17], but using a different emotion regulation scale (ERICA), the findings suggested that yoga has a significant effect on the emotion regulation capacities of adolescents that are not present in PE alone. Furthermore, as the semester progressed and the students experienced increases in demands such as exams and end-of-year projects, the PE group's emotion regulation decreased, perhaps providing further evidence as to the regulating benefits of the yoga intervention.

While it was predicted that the change scores in mindful awareness, self-compassion, and body awareness would correlate positively and significantly with the change scores in emotion regulation, the only significant correlation discovered was between body awareness and emotion regulation. Due to the nonsignificant correlations between all of the variables and emotion regulation, a mediation model could not be tested.

The correlation between body awareness and emotion regulation is an important, albeit preliminary, finding. The findings of this study showed a significant and positive correlation between body awareness and emotion regulation. This result may reflect how a practice such as yoga, with an emphasis on engagement with and awareness of the body, can be particularly effective with adolescents who are contending with multiple physical and physiological changes, all while developing a more firm sense of self and identification with their bodies [3]. This preliminary finding in adolescents mirrors studies with adults, which have found that increased body awareness leads to a sense of mastery and can impact self-regulatory skills [34, 35].

The nonsignificant findings regarding the relationships between self-compassion and mindful awareness with emotion regulation were not expected but may highlight important limitations posed by the developmental stage of middle adolescence (15–17 years old) [3, 4]. Although research with adults has supported the effects of self-compassion and mindfulness on self-regulatory skills and well-being [25, 26, 36], the findings may reflect the vulnerabilities that this population faces in regard to peer relationships, increased risk taking (i.e., impulsivity), and managing emotions [37]. Gilbert and Irons [38] discussed the difficult transition from childhood to adolescence and the increased focus on peer-group relationships, acceptance, and belonging. Often the emphasis is more on shame and self-criticism than self-compassion, particularly for those adolescents experiencing depressive symptoms or negativity [39]. Furthermore, while more maturation of the frontal lobe and stability of emotion regulation does not occur until about 18 years old [37], the middle adolescents in the current study may have not possessed the capacities to effectively learn and consistently utilize mindfulness skills. Others have theorized that increased awareness of negative affect and experiences may be overwhelming for youth and adolescents, leading to an avoidance of adopting mindfulness skills or a temporary increase in stress [23, 24, 40, 41]. Although middle adolescents are becoming more aware and self-sufficient, they are still very much limited in the amount of control they can exert over their day-to-day lives, possibly making increased awareness through mindfulness skills more frustrating than useful. The current findings may also support that interventions with the middle adolescent population either decrease the focus on mindfulness or explicitly confront coping with the inevitable negative emotions and experiences that will surface when increasing awareness.

Finally, given that there was a significant increase seen in emotion regulation for the yoga group, it is possible that other aspects of the practice contributed to this change but were not measured. Yoga is comprised of several components, and the effects of the breath work, relaxation, communication of the ethical principles, or a feeling of connectivity to the teachers and group cohesion, for example, may have also contributed to the results. Theoretically, the thinking around how yoga works to improve well-being is moving toward an attempt to understand the synergistic effects of its multiple aspects on cognitive, emotional, physical, and behavioral systems [42]. While this study did not show that the proposed mediating variables were partially responsible for the change in emotion regulation in this sample, it remains unclear if the results are due to specific aspects of this practice or a synergistic effect of the different aspects involved.

The current study utilized a strong methodology and a novel design; however there are several limitations to be noted. The study's small sample size dictates that all of the results be interpreted with caution. In addition to a lack of potential familiarity with the concepts being investigated, previous yoga studies with the adolescent population have questioned the reliability of student responses [43]. Participants were verbally encouraged to take their time and ask questions about any confusing or unclear items during scale administrations and efforts were made to remove data that was clearly unreliable. Although the scales were shown to be reliable and valid, it is deemed to be a limitation to the study that the student responses may have not been as thorough or as thoughtful as they could have been, and, consequently, the results may not fully represent the students' authentic experiences. Even though the class was required, attendance was low and inconsistent and may have impacted the correlations between the proposed mediating variables and the changes found in emotion regulation. Implementing an intervention within a public school, where students interact freely and have access to activities outside of school, prevented control for possible contamination across groups, and therefore the degree to which this occurred is unclear. A final limitation of the study is the homogeneity of the sample, limiting the generalizability of the results to other samples.

## 5. Conclusions

The current study provides initial evidence as to the positive effects of yoga on the emotion regulation of high school students. The results highlight potential benefits to emphasizing the physical aspects of yoga when working with this population. Replication or expansion of the current study would be ideal and valuable. Several questions remain regarding the mechanisms by which yoga impacts this self-regulatory capacity. Overall, there is still a need for methodologically strong studies with large sample sizes conducted in the school setting and with the adolescent population.

## Figures and Tables

**Table 1:** Participants' demographic information.

	Yoga (*n* = 19)	PE (*n* = 18)	Total sample mean or %	Statistics for group comparisons
Age	15 years = 4 16 years = 12 17 years = 1 Missing = 2	15 years = 4 16 years = 7 17 years = 1 Missing = 6	M = 16 years	*X* ^2^ = .47 *p* = .79

Sex	Male = 11 Female = 8	Male = 12 Female = 6	Male = 62.2% Female = 37.8%	*X* ^2^ * = * .30 *p* = .58

Ethnicity/race	Black = 17 Hispanic = 1 Other = 1 Missing = 0	Black = 15 Hispanic = 2 Other = 0 Missing = 1	Black = 86.5% Hispanic = 8.1% Other = 2.7% Missing = 2.7%	*X* ^2^ = 1.35 *p* = .51

Primary figure	Mother only = 1 Father only = 0 Mother and father = 15 Two fathers = 0 Missing = 3	Mother only = 4 Father only = 1 Mother and father = 7 Two fathers = 1 Missing = 5	Mother = 13.5% Father = 2.7% Mother and father = 59.5% Two fathers = 2.7% Missing = 21.6%	*X* ^2^ = 6.47 *p* = .09

Income	Below $10,000 = 2 $10–25,000 = 4 $25–50,000 = 3 $50–75,000 = 2 $75–100,000 = 3 $100–125,000 = 1 $125–150,000 = 0 Missing = 4	Below $10,000 = 2 $10–25,000 = 1 $25–50,000 = 1 $50–75,000 = 3 $75–100,000 = 1 $100–125,000 = 0 $125–150,000 = 1 Missing = 9	Below $10,000 = 10.8% $10–50,000 = 24.3% $50–100,000 = 24.3% Over $100,000 = 5.4% Missing = 35.1%	*X* ^2^ = 4.80 *p* = .57

Taking meds	Yes = 3 No = 15 Missing = 1	Yes = 0 No = 13 Missing = 5	Yes = 8.1% No = 75.7% Missing = 16.2%	*X* ^2^ = 2.40 *p* = .12

Attendance	Sessions attended M = 24.11	Sessions attended M = 22.61	Sessions attended M = 23.36	*t*(35) = .40 *p* = .69

Engagement 1 (week 8 of intervention)	Minimal = 0 Moderate = 0 Maximum = 13 Missing = 6	Minimal = 5 Moderate = 5 Maximum = 8 Missing = 0		*X* ^2^ = 10.66^*∗*^ *p* = .01

*Note*. ^*∗*^ *p* < .05 (2-tailed).

**Table 2:** Total sample means, standard deviations, and tests of normality.

Variable	*N*	M	SD	Skewness (SE)	Kurtosis (SE)
PRE (time 1)					
ERICA	37	55.38	6.42	.52 (.39)	.16 (.76)
MAASA	37	3.86	.73	−.35 (.39)	−.28 (.76)
SCS	36	79.33	14.22	−.09 (.40)	−.60 (.78)
MAIA	36	2.98	.60	−.02 (.39)	−.37 (.77)

POST (time 3)					
ERICA	34	56.32	8.20	.35 (.40)	1.12 (.79)
MAASA	34	3.89	.78	.79 (.40)	.16 (.79)
SCS	34	81.26	13.09	−.18 (.40)	−.29 (.79)
MAIA	33	3.13	.64	−.60 (.41)	.83 (.80)

Change scores					
ERICA	34	.82	6.17	−.16 (.40)	.07 (.79)
MAASA	34	.01	.86	.33 (.40)	−.52 (.79)
SCS	34	1.12	9.58	.51 (.40)	.61 (.79)
MAIA	32	.19	.64	−.61 (.41)	.39 (.81)

*Notes*. *N* = number of participants, M = mean, (SD) = standard deviation, and (SE) = standard error.

ERICA = Emotion Regulation Index for Children and Adolescents [27]; MAASA = Mindful Attention Awareness Scale in Adolescents [28]; SCS = Self-Compassion Scale [29]; and MAIA = Multidimensional Assessment of Interoceptive Awareness [30].
